# Intensive long-term pulmonary rehabilitation program after oesophagectomy, a reflection

**DOI:** 10.1186/2049-6958-7-22

**Published:** 2012-07-30

**Authors:** Franco Pasqua

**Affiliations:** 1 Department of Pulmonary Rehabilitation “IRCCS San Raffaele”, Rome, Italy

## There is body of evidence regarding the efficacy of Pulmonary Rehabilitation (PR) in the comprehensive management of patients with respiratory disease, and PR programs are practiced worldwide. Positive results in terms of improvement in dyspnoea, exercise capacity and Quality of Life (QoL) are recognized in Chronic Obstructive Pulmonary Disease (COPD). However PR is beneficial in other forms of chronic respiratory diseases, like asthma, cystic fibrosis, restrictive thoracic disease etc. Moreover, PR is becoming a crucial component of the overall treating strategy in high risk surgical patients (i.e. lung volume reduction surgery [LVRS] and lung transplantation), and in the pre- and post-operative period of patients who are candidates or have undergone lung resection (LR) for Non-Small Cell Lung Cancer (NSCLC).

Pulmonary function is commonly altered after surgery, particularly in patients with COPD, current o ex smokers, who have had chest or upper abdominal surgery. The physiological changes observed are directly related to anesthesia (general or regional) and to the type of incision and surgical technique employed, and are reflected by decreases in total pulmonary capacity and pulmonary volumes and by a parallel reduction of Pa0_2_. These functional alterations are in turn implicated in the development of postoperative pulmonary complications (PPC), which are the main causes of morbidity and mortality among surgical patients.

Therefore, the recovery of a good physical and respiratory function, the improvement of dyspnoea and quality of life are an important outcome in the postoperative management of these patients. Breathing exercises have commonly been used to increase pulmonary volumes and improve gas exchange and ventilation distribution. Diaphragmatic, segmental, and costal respiratory exercises, as well as sustained maximal inspiration, may alleviate surgically induced alterations such as diminished diaphragmatic mobility and restrictive pulmonary changes.

On the other hand a comprehensive program of pulmonary rehabilitation is able to improve dyspnoea, exercise capacity and quality of life of surgical patients, and it is now known that a better preoperative fitness is inversely related to postoperative complications. Also, the functional recovery in surgical patients is faster and complications are reduced after pulmonary rehabilitation.

Surgery for oesophageal cancer is a serious life-event, which may be accompanied by significant morbidity that may influence the patient’s quality of life. Common post-operative symptoms include dysphagia, weight loss and change in eating patterns. In addition, these patients experience, particularly in the first year after oesophageal resection, pain, fatigue, dyspnoea, depression and impaired quality of life, symptoms that are associated with increased risk of shorter survival. Among various postoperative complications after oesophagectomy, the pulmonary ones occur in approximately 30 % of cases and they are major causes of postoperative morbidity and mortality [1,2].

Despite these considerations, little evidence is currently available on the effectiveness of a comprehensive program of pulmonary rehabilitation on these symptoms, particularly the QoL, in subjects who have undergone surgery for oesophageal cancer [3].

The paper “Rationale and clinical benefits of an intensive long-term pulmonary rehabilitation program after oesophagectomy: preliminary report“, by Lococo and collegues, published in this journal, is interesting because it is, to our knowledge, the first devoted to this particular issue [4].

The authors retrospectively analyzed data from two groups of patients with a recent surgery for esophageal cancer, who underwent a standard rehabilitation therapy (control group) or a four weeks outpatient comprehensive program of pulmonary rehabilitation (treated group).

The results show a significant improvement in lung function (Total Lung Capacity, Forced Expiratory Volume and Forced Vital Capacity), in the treated group compared to the control group.

Similar improvements were found in terms of six minute walking distance (6MWD), dyspnoea, measured with the Visual Analog Scale (VAS) and Borg Scale, and Bartel Disability Index.

Considering that the deterioration of postoperative lung function is associated with a worse quality of life, the authors hypothesized that an improvement in lung function and exercise capacity may improve this outcome.

We think that the idea for the study is very interesting, but the methods and results are not clear. First, it is not acceptable that the authors did not use any questionnaire or investigations to measure QoL, while they did stress several times its role in patients who underwent oesophagectomy for cancer. Then, they do not clearly describe the rehabilitation protocol used in treated group; and finally the study was conducted retrospectively.

In our opinion, further prospectively planned studies would be necessary to investigate the effectiveness of pulmonary rehabilitation not only after, but also before the surgery, because pre-operative training programs may led to a reduction of hospital stay and post-operative complications.
